# HMD-Net: A Vehicle Hazmat Marker Detection Benchmark

**DOI:** 10.3390/e24040466

**Published:** 2022-03-28

**Authors:** Lei Jia, Jianzhu Wang, Tianyuan Wang, Xiaobao Li, Haomin Yu, Qingyong Li

**Affiliations:** 1 Beijing Key Lab of Traffic Data Analysis and Mining, Beijing Jiaotong University, Beijing 100044, China; 17112087@bjtu.edu.cn (L.J.); jzwang@bjtu.edu.cn (J.W.); xiaobaoli@bjtu.edu.cn (X.L.); 16120446@bjtu.edu.cn (H.Y.); 2 Shenzhen Urban Transport Planning Center Co., Ltd., Shenzhen 518000, China; uaing0515@gmail.com

**Keywords:** vehicles for hazmat transportation, hazmat marker detection, sparse regularization, channel pruning, YOLOv5, MobileNet

## Abstract

Vehicles carrying hazardous material (hazmat) are severe threats to the safety of highway transportation, and a model that can automatically recognize hazmat markers installed or attached on vehicles is essential for intelligent management systems. However, there is still no public dataset for benchmarking the task of hazmat marker detection. To this end, this paper releases a large-scale vehicle hazmat marker dataset named VisInt-VHM, which includes 10,000 images with a total of 20,023 hazmat markers captured under different environmental conditions from a real-world highway. Meanwhile, we provide an compact hazmat marker detection network named HMD-Net, which utilizes a revised lightweight backbone and is further compressed by channel pruning. As a consequence, the trained-model can be efficiently deployed on a resource-restricted edge device. Experimental results demonstrate that compared with some established methods such as YOLOv3, YOLOv4, their lightweight versions and popular lightweight models, HMD-Net can achieve a better trade-off between the detection accuracy and the inference speed.

## 1. Introduction

**Figure 1:**
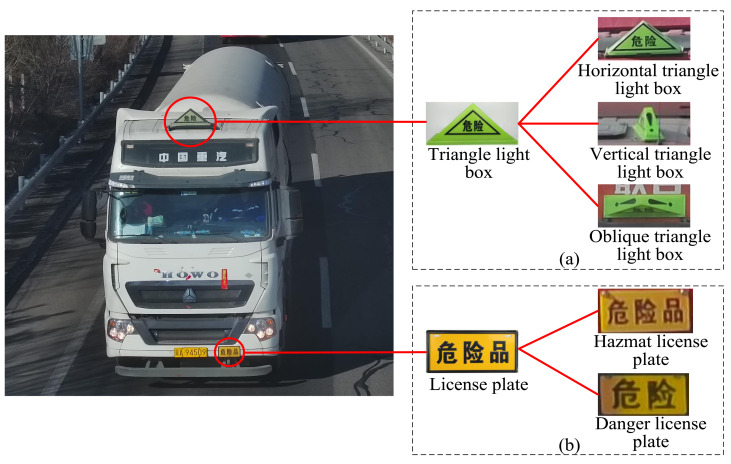
Examples of vehicle hazmat markers, which are generally installed or attached on the top and bottom front of the vehicle. (**a**) Triangle light boxes. (**b**) License plates.

Hazardous material (hazmat) transportation plays a crucial part for the development of economy and society, and it accounts for a large proportion of total highway transport. In general, hazmat refers to materials that are flammable and explosive, such as oxidizing substances and poisonous gases [1]. This means that once an accident occurs, vehicles for hazmat transportation could be very dangerous and likely to cause economic losses and even heavy casualties. Therefore, reducing the accident rate by detecting and monitoring vehicles for hazmat transportation is a common concern for the government, manufacturers and carriers. In fact, there are many ways for vehicle monitoring [2,3]. In terms of vehicles for hazmat transportation, as required by the Standardization Administration of China, they must be installed and attached with corresponding warning markers, including triangle light boxes and license plates, as shown in Figure 1a and Figure 1b, respectively. Consequently, recognizing vehicles for hazmat transportation by detecting hazmat markers becomes a natural fit. Hence, computer vision based hazmat marker detection has attracted more and more attention.

Traditional visual methods for hazmat marker detection mainly rely on hand-crafted features. For instance, Gossow et al. [4] combined histogram backprojection and speeded up robust features (SURF) and presented a danger sign detection method, but the detection accuracy remains to be improved. Ellena et al. [5] quantified the characteristics of hazard markers and compared them with a candidate picture for hazmat sign detection. Based on the geometric onstraints and saliency maps, Parra et al. [6] introduced two hazmat sign recognition methods. Based on character-specific extremal regions, Gou et al. [7] proposed a vehicle license plate recognition method by hybrid discriminative restricted Boltzmann machines. Owning to the issues of feature robustness, the performance of these types of methods is usually not satisfactory enough, and it is difficult to adapt them to various environments, such as unstable lighting conditions, inconstant imaging angles and intense contrast changes.

With the development of convolutional neural networks (CNN), a variety of deep learning based methods have been put forward for hazmat marker detection. Considering the demand in accuracy and efficiency, Sharifi et al. [8] designed a deep learning based hazmat sign detection robot with restricted computational resources. Based on a cascaded network, Wang et al. [9] proposed a light convolutional neural network for license plate detection and recognition with higher accuracy and lower computational cost. To improve the detection performance of CNN based models, Cai et al. [10] tried to rectify images with geometric information to compensate for the perspective distortion before feeding them to the detector module. Xie et al. [11] proposed a CNN-based framework for multi-directional car license plate detection. To solve the problem of object detection under low illumination, Li et al. [12] proposed an illumination-aware Faster R-CNN (IAF R-CNN). An illumination-aware network is introduced to give an illumination measure of the input image. Zhuang et al. [13] proposed a lightweight illumination and temperature-aware multispectral network for accurate and efficient pedestrian detection. On adverse weather conditions, Sindagi et al. [14] proposed an unsupervised prior-based domain adversarial object detection framework for adapting the detectors to hazy and rainy conditions.

Though deep learning-based methods often have superior performance than traditional methods, they still face several challenges. Firstly, training a deep learning-based model usually requires a large number of annotated images, but there is not a public dataset for hazmat marker detection. On the one hand, collecting dataset for research is a time-consuming process; on the other hand, the quality and accuracy of the collected hazmat data are affected by the skill and alertness of the data collectors, as proposed in [15]. Secondly, CNN mostly involve in heavy computation and deep learning-based models are usually computationally expensive. Sharifi et al. [16] proposed a CNN-Based pipeline called DeepHAZMAT for detecting and segmenting hazmats. The DeepHAZMAT network had to cope with some challenges such as restricted CPU and computational resources which are embedded in a rescue robot. Due to the limited hardware resources on edge devices, it is difficult to deploy the model on hardware. Considering the factors reported above, we aim to provide a benchmark for vehicle hazmat marker detection. The main contributions are summarized as follows:

1. We release a large-scale vehicle hazmat marker dataset, which contains hazmat marker images captured under complex environmental conditions in a real expressway. To the best of our knowledge, this is the first open-sourced hazmat marker detection dataset.
2. We build a hazmat marker detection network named HMD-Net, combining the merits of the cutting-edge detector and lightweight backbone, which help to achieve a trade-off between the accuracy and efficiency.
3. We implement sparse training for the network and further compact the model by channel pruning operations, which enable the success of deploying the model on a resource-restricted edge device for real-time vehicle hazmat marker detection.

The rest of this paper is organized as follows. We review some preliminaries in Section 2. Section 3 details the proposed hazmat marker detection network. In Section 4, experiments on the released real-world hazmat marker detection dataset are conducted and analysed, followed by conclusions and future works in Section 5.

## 2. Preliminaries

### 2.1. Object Detection

According to whether region proposals are needed, deep learning based object detection can be roughly divided into two-stage and one-stage methods.

Two-stage methods usually first generate region proposals and then refine them to obtain final results. Typically, Girshick et al. [17] combined CNN features with region proposals (R-CNN) to detect objects, but the efficiency is not satisfactory. In response to this, He et al. [18] integrated spatial pyramid pooling (SPP) in CNN, which significantly improves the detection speed while remains the detection accuracy. Afterwards, Girshick et al. [19] proposed a Fast R-CNN, employing a softmax classifier and region proposal strategy to help achieve end-to-end training. However, the selective search procedure in Fast R-CNN significantly limits the detection speed. To this end, Ren et al. [20] replaced the selective search with a region proposal network (RPN), which dramatically improves the detection speed. Overall, although two-stage detection methods have achieved great success, most of them still face the problem of high computation cost and slow detection speed, and all these factors remarkably affects their applications in real scenarios.

To avoid the above-mentioned problems, many attempts have been made to achieve one-stage object detection. Initially, Redmon et al. [21] formalized object detection as a regression problem of spatially separated bounding boxes and associated class probabilities, and proposed a model called YOLO, meaning ‘you only look once’. Subsequently, YOLOv2 [22] was presented, which takes Darknet19 as the backbone and adopts operations like batch normalization(BN) [23] and high resolution classifier to improve the detection performance. Then, YOLOv3 [24] was presented, replacing the backbone with Darknet53 and integrating the feature pyramid network (FPN) [25]. To date, the detection accuracy has been improved a lot, but the speed is still a bottleneck. More importantly, it is not optimized for detecting small objects. To this end, YOLOv4 [26] and YOLOv5 (https://github.com/ultralytics/yolov5 (accessed on 1 February 2022)) were successively proposed, in which the backbones are specially optimized and augmentations like mosaic are adopted to boost the detection performance. In addition, the path aggregation network (PANet) [27] instead of FPN is used for parameter aggregation. More importantly, YOLOv5 utilizes an adaptive anchor, which is beneficial for small target detection. Consequently, YOLOv5 is employed as the basic network in our work.

### 2.2. Lightweight Models

In recent years, deep learning based models have achieved great success in a variety of domains. However, on the one hand, these methods indeed show good performance in specific tasks, but on the other hand, they usually have fair-sized volumes and make a large amount of calculations, which make them hard to work on resource-restricted hardwares. Therefore, how to obtain compact and efficient models is essential for real deployment, and one solution is to train lightweight models by designing specific network architectures.

In order to deploy a deep model on mobile and embedded devices, Howard et al. [28] put forth a new network architecture, i.e., MobileNet. It replaces standard convolution operations with depth-wise separable convolutions and involves in two global hyper-parameters to balance the accuracy and latency. To avoid the vanishing gradient problem, Sandler et al. [29] put forward MobileNetV2 by introducing the inverted residual block, where depth-wise operations are added to lower the memory requirement during the inference stage. In addition, non-linear layers are removed in the bottleneck to help maintain the feature diversity and further enhance the representation ability. Afterwards, technologies like neural architecture search and attention mechanism were exploited in MobileNetV3 [30]. However, its detection speed is somewhat reduced compared to MobileNetV2. Taking into account our actual demands in hazmat marker detection, a revised MobileNet is utilized to serve as the backbone of in our work.

### 2.3. Network Pruning

As mentioned above, training lightweight models is a feasible way to achieve model compactness and efficiency. However, in fact, one can further compress a pre-trained model with particular implementation strategies.

Decomposition, quantification and knowledge distillation have been considered as three effective means for model compression and acceleration. Typically, the singular value decomposition was adopted in [31] to to reduce unnecessary calculations by approximating the weight matrix, but it essentially does not speed up the convolutions. Courbariaux et al. [32] provided a weight binarization solution to convert the original multiplication operations into addition or shift operations, which are expected to improve the inference speed. However, it is easy to yield the training difficulty and accuracy reduction. Hinton et al. [33] introduced the way that distilling the knowledge in an ensemble of models into a single model that is much easier to deploy. Unfortunately, it implicitly requires that teacher networks can describe the whole data distribution, which may not always hold in practice.

Nowadays, network pruning is considered as one of the most promising techniques for model compression and acceleration. Compared with other techniques, it intends to directly remove the redundant weights from the network. According to pruning granularity, it can be divided into unstructured pruning and structured pruning. The former refers to prune the individual weights, while the latter usually implements filter-wise or channel-wise pruning. For example, LeCun et al. [34] initially presented the optimal brain damage, an unstructured prune strategy using the second-order derivative information to achieve a trade-off between the network complexity and training error, but it does not accelerate the training process. In contrast, Li et al. [35] proposed to prune channels with small incoming weights in training CNN, and then fine-tune the network to regain accuracy. Compared with these works, Liu et al. [36] explicitly imposed channel-wise sparsity in the optimization objective during training, yielding smoother channel pruning process and little accuracy degradation. Thus, it is adopted in this work.

## 3. Hazmat Marker Detection Network

**Figure 2:**
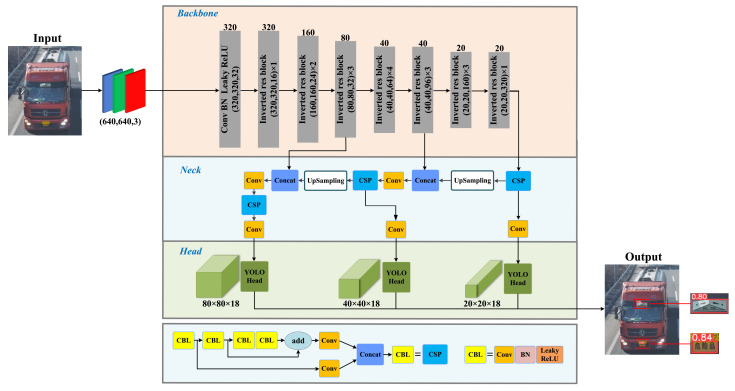
The overall framework of HMD-Net. CBL is the foundation of our network structure and CSP is the abbreviation of the cross stage partial network.

Figure 2 illustrates the overall framework of the proposed HMD-Net, and the critical details including network architecture, loss functions and network pruning are elaborated in the following subsections.

### 3.1. Network Architecture

Based on the demand for good detection accuracy, relatively few network parameters and fast inference speed, the proposed HMD-Net as a whole follows the architecture design of well-established YOLO series. HMD-Net applies some optimization in some aspects. Specifically, it can be divided into three parts, namely, the backbone, neck and head networks.

The backbone network is responsible for extracting image features and transmitting them to the neck module. To further compress the model, we implement a lightweight backbone based on MobileNetV2, which integrates a reverse residual structure with depth-wise separable convolution. The improved MobileNetV2 significantly decreased the number of operations and memory needed while retaining the accuracy at the same level.

More specifically, compared with the raw MobileNetV2 that has 19 bottlenecks, the improved MobileNetV2 uses 12 fewer bottlenecks, and eliminates the focus module and SPP module than the cross stage partial network (CSPNet) [37]. Each bottlenecks contains three convolutional layers, of which the second layer adopts the 3×3 depth-wise separable convolution, while the others are general 1×1 convolutions. The intermediate expansion layer uses lightweight depthwise convolutions to filter features as a source of non-linearity. In addition, except for the last convolutional layer, all other convolutional layers are implemented with BN and ReLU6 activation [29] during the training process.

The neck network fuse and concatenate multi-scale features to enhance the detection performance for varisized vehicle hazmat markers. In particular, the FPN and PANet are successively used for transferring robust semantic features and positioning features from the top to bottom. Among the neck network, the CSPNet is able to divide the features of the primary layers into two parts and then combine them through a cross-stage hierarchical structure, reducing the amount of calculation and ensuring the accuracy.

Based on the multi-scale features from the neck network, the head network uses the anchors to generate the prediction frame with the category, coordinate and confidence information. In order to be better prepared for multi-scale markers, the proposed HMD-Net has three heads, receiving feature maps of different sizes. In implementation, the sizes of input feature maps are set to 80×80, 40×40 and 20×20, respectively.

### 3.2. Loss Functions

In the training phase, it is inevitable for the network to output some prediction boxes that do not contain any hazmat markers. To this end, the confidence loss function is adopted to help remove those false positives. Specifically, it is a binary cross entropy loss function, which is formally defined as:

(1) $$L_{Conf}\;=\;\;-\;\sum_{i=0}^{S^{2}}\sum_{j=0}^{B}\left[{\hat{C}}_{i,j}\log\left(C_{i,j}\right)\;+\;\left(1\;-\;{\hat{C}}_{i,j}\right)\log\left(1\;-\;C_{i,j}\right)\right]$$

where $S^{2}$ is the number of grid cells, *B* denotes the number of prior anchors in each grid, ${\hat{C}}_{i,j}$ represents the intersection of union (IoU) [38] between the predicted bounding box and the ground-truth, and $C_{i,j}$ signifies the confidence that there is a hazmat marker in the predicted bounding box.

In order to further refine the position of the prediction box, a complete IoU(CIoU) loss function [39] is involved in the training process. Compared with other IoU based loss functions, CIoU introduces an aspect ratio to solve the problem of center point overlapping [39]. Mathematically, it is defined as:

(2) $$L_{CIoU}=1-IoU+\frac{{æ}^{2}\left(b,b^{gt}\right)}{c^{2}}+\alpha \cdot \nu$$

where IoU measures the intersection of the union between the prediction box and the ground-truth, $\rho^{2}\left(\cdot \right)$ computes the Euclidean distance between two central points *b* and $b^{gt}$ of the predicted bounding box and the ground-truth, and *c* denotes the diagonal length of the smallest enclosing box covering the two boxes. ν measures the consistency of aspect ratio and is defined as:

(3) $$\nu =\frac{4}{\pi^{2}}{\left(arctan\frac{w^{gt}}{h^{gt}}-arctan\frac{w}{h}\right)}^{2}$$

where (w,h) and $\left(w^{gt},h^{gt}\right)$ are the width and height of the predicted bounding box and the ground-truth, respectively. As a trade-off parameter, α is usually defined as:

(4) $$\alpha =\frac{\nu }{(1-IoU)+\nu }$$

To prepare for channel pruning, we integrate a sparse regularization module, which functions is based on the BN process. The BN layer is usually embedded behind the convolutional layer and able to address the problem of internal covariate shift by normalizing layer inputs, which can be formalized as:

(5) $$y=\gamma \hat{x}+\beta ,with\;\hat{x}=\frac{x-\mu_{B}}{\sqrt{\sigma_{B}^{2}+\epsilon }}$$

where *x* is the input of a BN layer, and $\hat{x}$ represents the z-score result of *x*, $\mu_{B}$ and $\sigma_{B}^{2}$ denote the mean and variance of the input features, respectively. γ and β are the scale factor and deviation that need to learned from the training procedure. Herein we use γ as a measure of channel importance and define a regularization loss as:

(6) $$L_{Reg}=\sum_{\gamma \in \Gamma }g\left(\gamma \right)$$

where Γ denote the set of scale factors and g(·) is implemented as $\ell_{1}$-norm to help pursue the channel sparsity.

Considering that our goal is to detect vehicle hazmat markers, no matter what categories the markers are. Therefore, traditional classification based loss functions are not utilized in our work. Overall, the final loss function for HMD-Net can be formalized as:

(7) $$L=L_{Conf}+L_{CIoU}+\lambda \cdot L_{Reg}$$

where λ is the trade-off parameter to balance the regularization loss with other losses. It should be mentioned that, in order to better perform channel pruning as described in the next subsection, the sparse regularizer, namely, $L_{Reg}$, works if and only if HMD-Net has been trained for 100 epochs. In other words, λ is set to zero when the number of epochs is less than 100. When it begins to play a role, λ is set to $6\times 10^{-4}$.

### 3.3. Channel Pruning

After trained on the open-sourced VisInt-HMD dataset, the proposed HMD-Net has been able to effectively detect vehicle hazmat markers. However, a cumbersome number of network parameters, on the one hand, help the HMD-Net to achieve a satisfactory detection performance, but on the other hand, they will cause much redundant or even unnecessary computations and further hinder the model from being easily deployed on resource-restricted devices. Actually, there is still plenty of room for reducing the number of model parameters. Therefore, it is essential to carry out the pruning process for the model.

As the mainstream technology, channel pruning intends to remove superfluous network channels based on their importance. As pointed out in [36], the absolute values of γ in BN layers can be seen as an importance measurement of the corresponding channels. After the sparse training process, the values of unimportant channels will approach to zero. In other words, the channels with higher γ values are deemed to be essential and thus should be retained, whereas those with smaller γ values are less important and can be pruned. In this way, we can reduce the number of network parameters, decrease the calculation amount and lower the hardware requirements. Obviously, the pruning process will effect the detection accuracy, and it is necessary to further fine-tune the pruned model for recovering its detection capacity.

## 4. Experiments

### 4.1. VisInt-VHM Dataset

To verify the effectiveness of the proposed method, we collect and release a real-world vehicle hazmat marker dataset (The dataset is open-sourced at: https://github.com/jialei-bjtu/VisInt-VHM.git, 1 February 2022) and the details are as follows.

To ensure the usability and reliability of the collected data, one Hikvision monitoring camera (iDS-TCV900-AE/25) is deployed at the entrance of Taijia Expressway in Shanxi province in China for image capturing. This camera is installed on the roadside pole with a height of 5.8 m and uses the infrared flash as the supplementary lighting. The captured images cover two lanes of the expressway, with the resolution being 4096×2160. All images are captured during the period of November 2019 to April 2020.

**Figure 3:**
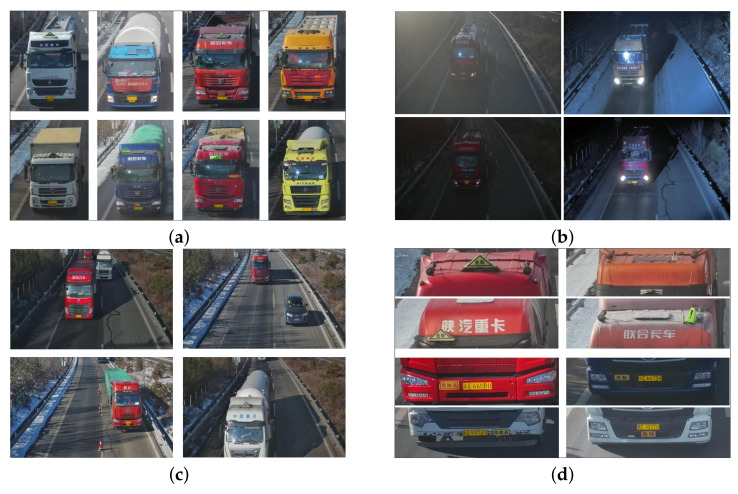
Examples of different hazmat vehicle marker in VisInt-VHM Dataset. (**a**) The appearance of different hazmat vehicles. (**b**) Images captured under different weather and light conditions. (**c**) Images captured under different shooting angles. (**d**) Images of hazmat markers fixed irregularly.

Quantitatively, we have collected 10,000 images of front-view hazmat vehicles, considering three aspects of variations. Firstly, there are totally 24 kinds of vehicles with diverse appearances, as shown in Figure 3a. Secondly, these images are captured under different weather and light conditions, which may severely degenerate the quality of resulting images, as shown in Figure 3b. Thirdly, it is very likely that images are captured with dynamic shooting angles, which also poses some difficulty for hazmat marker detection, as shown in Figure 3c. These vehicle images totally consist of 20,023 hazmat markers, including triangle light markers and license plates with different postures and positions, as shown in Figure 3d. All markers in images are annotated with bound-boxes by LabelImg (https://github.com/tzutalin/labelImg, 1 February 2022). It should be noted that though there are a wide range of variations about these images, there is only one kind of label for them, since our focus is vehicle hazmat marker detection rather than classification.

### 4.2. Baseline Methods

We compare the proposed HMD-Net with the following baseline methods:

- YOLOv3 [24]: It replaces the backbone of the initial YOLO with Darknet53 and predicts an objectness score for each bounding box using logistic regression. It extracts features from those scales using a similar concept to feature pyramid networks for multi-scale fusion, which help to detect objects of different sizes and thus significantly improve the detection accuracy.
- YOLOv3-Tiny (https://github.com/AlexeyAB/darknet, 1 February 2022): As a tiny version of YOLOv3, the backbone network uses a 7-layer convolution and Maxpool to extract features, and only retains two independent prediction branches with resolutions of 13×13 and 26×26.
- YOLOv4 [26]: The backbone of YOLOv4 is CSPDarknet53, which contains 29 convolutional layers 3 × 3, a 725 × 725 receptive field and 27.6M parameters. It uses PANet as the method of parameter aggregation from different back- bone levels for different detector levels, instead of the FPN used in YOLOv3.
- YOLOv4-Tiny (https://github.com/AlexeyAB/darknet, 1 February 2022): As a small version of YOLOv4, it simplifies the backbone network of YOLOv4, uses leaky ReLU as the activation function, and removes the SPP module. In addition, FPN is used to extract feature maps of different scales, and only two prediction branches are retained.
- YOLOX-S [40]: YOLOX enables the detector of YOLO to work in an anchor-free manner and integrates some tricks like mixup augmentation [41] and optimal transport assignment [42] to further improve the detection performance. Based on YOLOX, YOLOX-S shrinks the number of channels by a large margin and serves as a small version of the standard models of YOLOX series.
- YOLOX-Tiny [40]: As a tiny version of YOLOX, it removes the hybrid enhancement and weakens the mosaic in the training phase. Compared with Yolov4-Tiny, the detection performance of Yolox-Tiny is significantly improved when the number of parameters is reduced by 1 M.
- YOLOX-Nano [40]: As a nano version of YOLOX, it follows the basic structure of YOLOX-Tiny but further simplifies the backbone network, making it can be easily deployed on mobile devices.
- YOLO-ResNet50 [43]: We replace the backbone network of YOLOv5 with Resnet50 and named YOLO-ResNet50. These residual networks are easier to optimize, and can gain accuracy from considerably increased depth.
- YOLO-MobileNetv2 [29]: We replaced the backbone network of YOLOv5 with the raw MobileNetv2 and named YOLO-MobileNetv2, which integrates a reverse residual structure with depth-wise separable convolution. MobileNetv2 is a neural network architecture that is specifically tailored for mobile and resource constrained environments.

### 4.3. Evaluation Metrics

Since HMD-Net undertakes the task of detection, the average precision (AP) is taken as the main evaluation metric to compare the proposed HMD-Net with the baselines, which is defined as:

(8) $$AP=\int_{0}^{1}P(R)dR$$

where *P*, *R* denotes the precision and recall, respectively, and can be computed by:

(9) $$P=\frac{TP}{TP+FP}\times 100\%$$

(10) $$R=\frac{TP}{TP+FN}\times 100\%$$

where TP is the number of correctly detected hazmat markers, FP denotes the number of regions that are falsely marked as hazmat markers, and FN presents the number of undetected hazmat markers. It should be explained that in our case, the detection is correct if and only if the IoU between the predicted bounding box and ground-truth is larger than 0.5. In addition, the number of floating-point operations (FLOPs) and network parameters are adopted as measures for model complexity. In order to further evaluate models’ usability on resource-restricted devices, the frame per second (FPS) is used for measuring the detection speed. For AP and FPS, the larger the values, the better performance the model has. As for FLOPs and parameters, the situation is just the opposite, i.e., the smaller the values, the more compact and efficient the model is.

### 4.4. Implementation Details

Without loss of generality, all training and testing images are resized to 640×640. There are 7425 and 2574 images in the training and testing sets, respectively. All experiments are conducted on a server with Intel Core i9-10900K CPU, 32 GB RAM and NVIDIA RTX 3080 GPU. As for HMD-Net, it is trained using the stochastic gradient descent (SGD) optimizer with a learning rate of 0.01 and a batch size of 16 for 500 epochs. In the first 100 epochs, only the confidence loss and CIoU loss are adopted, which aims at improving the detection performance of the basic model. In the following 200 epochs, the regularization loss begins to work, leading to sparse model weights and thus facilitating the pruning process. After pruning 70% channels of the HMD-Net, we further fine-tune it for 200 epochs, which is expected to recover the detection capacity of the pruned model. Except for the above-mentioned parameters, the remaining parameters follow the default settings of YOLOv5.

### 4.5. Performance Comparison

Quantitative results of different methods on VisInt-VHM dataset are shown in Table 1. Since less simplifications are adopted in YOLOv3, it has the highest FLOPs among all the methods, which means a computation-intensive procedure in the inference stage. After removing some feature layers of the backbone and only retaining two independent prediction branches, YOLOv3-Tiny significantly reduces the FLOPs with little performance degradation. Similar conclusions can be drawn for YOLOv4 and YOLOv4-Tiny. However, YOLOv4 integrates more tricks for object detection and achieves higher values of AP. However, at the same time, it has more parameters than YOLOv3. Different from YOLOv3-Tiny, the performance of YOLOv4-Tiny is less satisfactory, indicating that operations like removing the PANet in the neck network seriously affect the usability of the model.

We compare the detection performance of two lightweight models YOLO-ResNet50 and YOLO-MobileNetv2. YOLO-ResNet50 has similar detection performance to YOLOv3 and YOLOv4. The parameters of YOLO-ResNet50 are less than half of YOLOv3 and YOLOv4, and the FLOPs is about half of them. The parameters and the FLOPs of YOLO-MobileNetv2 are smaller than those of YOLOv3-tiny and YOLOv4-tiny, but its AP is equivalent to that of YOLOv3 and YOLOv4.

YOLO-MobileNetv2 and HMD-Net have very similar network architecture. The parameters and FLOPs of YOLO-MobileNetv2 are more than 5 times that of HMD-Net. The backbone of HMD-Net is an improved MobileNetv2 with 12 bottlenecks, less than the raw MobileNetv2 with 19 bottlenecks. Furthermore, we implement sparse training for HMD-Net and further compact the model by channel pruning operations. Therefore, the similar structures models have great differences in parameters and FLOPs.

Although YOLOX series has proved its success in object detection, its ability in detecting vehicle hazmat markers leaves much to be desired. Specifically, the AP only reaches 90.88%, which is the worst performance in comparison methods. YOLOX series are anchor-free detectors, which significantly reduce the number of design parameters and make the model considerably simpler [40]. However, anchor-free detectors cause label noise during training, since some of these positively labeled features may be on the background or an occlude object [44]. It reduces the performance of the model in small object detection. Based on VisInt-HMD dataset, the hazmat marker detection is a typical small object detection scenario. Therefore, the experimental performance of YOLOX series models is not ideal.

The proposed HMD-Net achieves an AP of 99.35%, comparable to YOLOv3, YOLOv4, YOLO-ResNet50 and YOLO-MobileNetv2. However, in the meanwhile, due to the sparse training and channel pruning, HMD-Net has the smallest number of parameters and FLOPs, namely, 0.29M parameters and 1.2G FLOPs. In other words, the proposed HMD-Net is able to achieve a good balance between the detection accuracy and computation amount.

### 4.6. Effectiveness Verification of Sparse Regularizer

**Figure 4:**
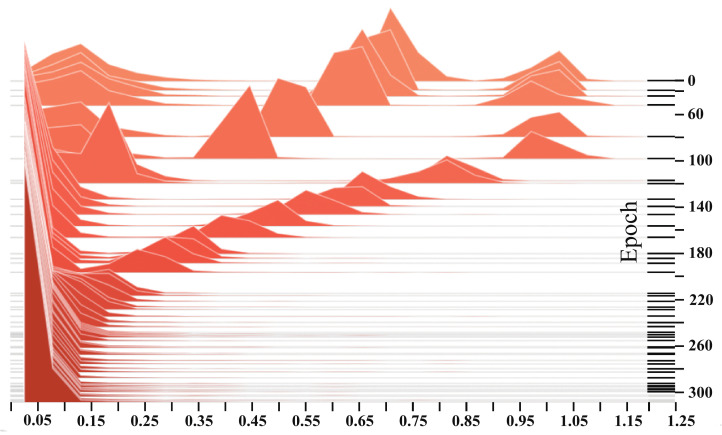
Statistics of scaling factors in all BN layers during the training process of HMD-Net.

To verify the effectiveness of the sparse regularizer, we visualize the histograms of the values of γ in all BN layers with the increasing epochs during the training process. It can be seen in Figure 4 that in the first 100 epochs, there does not seem to be a clear distribution pattern for the values of γ. In contrast, there are many values that are close to 1. When $L_{Reg}$ in Equation (7) begins to work, that is, the epoch index is larger than 100, we can find that more and more γ values are becoming smaller and gradually approaching 0, showing a sparse distribution. After training 300 epochs, there are only slight changes in the distribution of γ values and most of the values are close to 0, which indicates that the sparse training has been saturated. This reflects that the sparse regularizer indeed has an impact on the distribution of model weights, making the channels of convolutional layers become sparse and thus facilitating the pruning process.

### 4.7. Deployment and Analysis

As reported in the last subsection, HMD-Net has the smallest number of parameters and FLOPs, indicating its potential application in real-world hazmat marker detection scenarios. To verify the feasibility of applying the HMD-Net in practical scenes, we resort to a resource-restricted device and analyze the results by changing the pruning rates and ablation studies.

#### 4.7.1. Deployment

**Figure 5:**
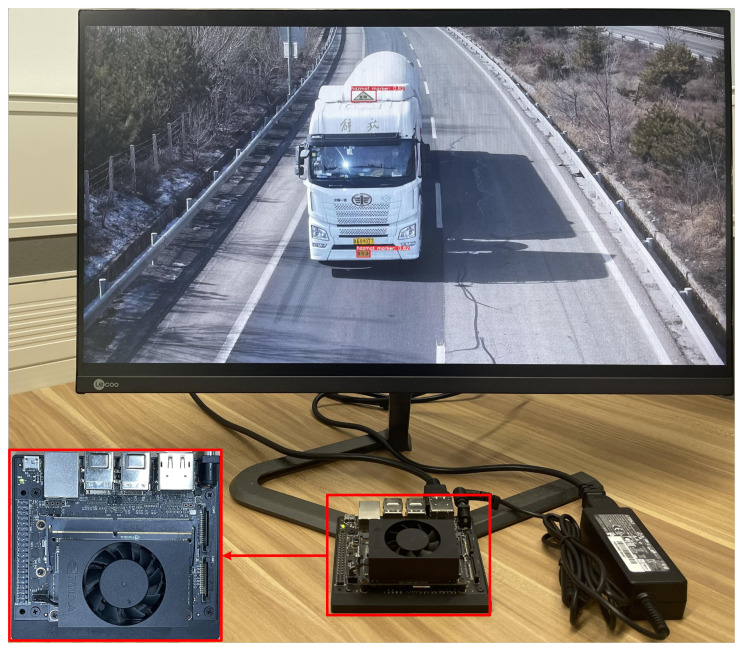
The actual deployment of HMD-Net on Jetson Xavier NX.

In this paper, Jetson Xavier NX, a GPU-based edge computing device released by NVIDIA is adopted. It has 384 CUDA cores, 48 Tensor cores and 2 NVIDIA deep learning accelerator (NVDLA) engines. It is able to run multiple modern neural networks in parallel and process high-resolution data from multiple sensors simultaneously. In addition, Jetson Xavier NX can provide at most 21 tera operations per second (TOPS) computation capacity. All of these factors make it a good choice for testing the usability of a pre-trained model. Just as expected, after utilizing the revised lightweight backbone and implementing effective channel pruning process, we have successfully deployed the proposed HMD-Net on this resource-restricted device, as shown in Figure 5. More performance analysis on this device are followed in the next two subsections.

#### 4.7.2. Effect of Different Pruning Rates

In order to analyze the effect of the pruning rate, we here additionally set two different pruning rates of 60% and 80% for HMD-Net. As shown in Table 2, HMD-Net with the pruning rate of 60% achieves the highest AP, but also requires the most parameters and FLOPs, making it has the slowest detection speed. In contrast, HMD-Net with the pruning rate of 80% enjoys the lowest parameters and FLOPs and thus has the fastest detection speed, but its AP decreases by 1%, which will cause more false positives. Therefore, it is observed the pruning rate has a direct impact on HMD-Net, and the default pruning rate of 70% indeed helps HMD-Net achieve a balance between the accuracy and speed and obtain an overall optimal performance.

#### 4.7.3. Results of Ablation Studies

Since HMD-Net as a whole follows the architecture design of YOLOv5, we conduct ablation studies with the following three settings:

- YOLOv5-S: YOLOv5 with the smallest backbone that is widely deployed on edge devices.
- HMD-Net without pruning: YOLOv5 with our revised MobileNetV2 as the backbone.
- HMD-Net: YOLOv5 with our revised MobileNetV2 as the backbone and pruning.

As shown in Table 3, as the smallest version of YOLOv5, YOLOv5-S achieves the highest AP of 99.46%, but its detection speed is less satisfactory. By only replacing the backbone with our revised MobileNetV2, HMD-Net without pruning significantly reduces the number of parameters and FLOPs by 83.94% and 80%, respectively, which proves the importance of the revised MobileNetV2. After channel pruning process, HMD-Net obtains the lowest number of parameters and FLOPs and highest FPS of 38 f/s, which can basically satisfy the requirement for real-time vehicle hazmat detection.

## 5. Conclusions and Future Works

In this paper, we release a large-scale high-quality data set, named VisInt-VHM for vehicle hazmat marker detection. This dataset contains 10,000 images of front-view hazmat vehicles, considering three aspects of variations, i.e., appearance of vehicle, light conditions and dynamic shooting angles. VisInt-VHM totally consists of 20,023 hazmat markers, including triangle light markers and license plates. In addition, we provide a lightweight vehicle hazmat marker detection model, i.e., HMD-Net, which uses our revised MobileNetV2 as the backbone and integrates channel pruning operations, and all these attempts finally help us successfully deploy the proposed HMD-Net on a resource-restricted edge device. Experimental results show that the proposed HMD-Net is able to achieve a good balance between the detection accuracy and speed.

In future work, we plan to extend the research in two aspects. Firstly, based on the detection and discrimination of hazmat vehicles, we will study optical character recognition to determine the types and quantities of hazardous ma terials being transported to ensure timely response to hazmat incidents. Secondly, We hope to employ infrared cameras to collect the datasets on low-illumination conditions and to research multispectral networks in order to cope with the effects of low-illumination conditions.

## Figures and Tables

**Table 1:** Performance comparison of different methods.

Algorithms	AP (%)	Parameters (M)	FLOPs (G)
YOLOv3	99.41	78.72	154.57
YOLOv3-Tiny	98.81	8.67	12.91
YOLOv4	99.48	81.03	140.98
YOLOv4-Tiny	96.62	5.87	16.14
YOLOX-S	90.88	8.94	26.64
YOLOX-Tiny	90.89	5.03	15.14
YOLOX-Nano	90.86	2.24	6.87
YOLO-ResNet50	99.55	27.85	75.9
YOLO-MobileNetv2	99.39	3.56	6.6
**HMD-Net**	99.35	**0.29**	**1.2**

**Table 2:** Performance comparison of HMD-Net with different pruning rates.

Model (Pruning Rate)	AP (%)	Parameters (M)	FLOPs (G)	FPS (f/s)
HMD-Net (60%)	99.43	0.42	1.4	37
HMD-Net (80%)	98.53	0.13	0.8	39
HMD-Net (70% by default)	99.35	0.29	1.2	38

**Table 3:** Performance comparison of HMD-Net and YOLOv5-S of lightweight backbone network.

Model	AP (%)	Parameters (M)	FLOPs (G)	FPS (f/s)
YOLOv5-S	99.46	6.85	11.5	32
HMD-Net without pruning	99.43	1.10	2.3	35
HMD-Net	99.35	0.29	1.2	38
